# Electron generation in water induced by magnetic effect and its impact on dissolved oxygen concentration

**DOI:** 10.1186/s42834-021-00080-0

**Published:** 2021-02-10

**Authors:** Augustine Chung Wei Yap, Hwang Sheng Lee, Joo Ling Loo, Nuruol Syuhadaa Mohd

**Affiliations:** 1 Department of Mechanical and Material Engineering, Universiti Tunku Abdul Rahman, 43000 Kajang, Malaysia; 2 Centre for Photonics and Advanced Materials Research, Universiti Tunku Abdul Rahman, 43000 Kajang, Malaysia; 3 Department of Mechatronics and Biomedical Engineering, Universiti Tunku Abdul Rahman, 43000 Kajang, Malaysia; 4 Department of Civil Engineering, University of Malaya, 50603 Kuala Lumpur, Malaysia

**Keywords:** Electron density, Dissolved oxygen concentration, Magnetic effect, Reversed electric motor principle, Free radical, Water quality parameters

## Abstract

pH, oxidation-reduction potential (ORP) and dissolved oxygen (DO) concentration are important parameters in water quality surveillance and treatment. The changes of these parameters are associated with electron density in water. Several techniques including electrolysis and catalysis which require redox reactions and electron exchange are employed to improve these parameters. In recent years, studies reported that magnetic effects can impart considerable changes on the pH, ORP and DO concentration of water. However, the correlation between electron density and magnetic effects on these parameters has yet to be disclosed despite the fact that increased electron density in water could improve water’s reductive properties, heat capacity and hydrogen bonding characteristics. In this study, the magnetic effects on pH, ORP and DO concentration were investigated using different magnets arrangements and water flow rates based on reversed electric motor principle. Results showed that the improvement of pH, ORP and DO concentration from 5.40–5.42 to 5.58–5.62 (+ 3.5%), 392 to 365 mV (− 6.9%), and 7.30 to 7.71 mg L^− 1^ (+ 5.6%), respectively were achieved using combined variables of non-reversed polarity magnet arrangement (1000–1500 G magnetic strength) and water flow rate of 0.1–0.5 mL s^− 1^. Such decrement in ORP value also corresponded to 8.0 × 10^13^ number of electron generation in water. Furthermore, Raman analysis revealed that magnetic effect could strengthen the intermolecular hydrogen bonding of water molecules and favor formation of smaller water clusters. The findings of this study could contribute to potential applications in aquaculture, water quality control and treatment of cancer attributed to free radical induced-oxidative stress.

**Supplementary Information:**

The online version contains supplementary material available at 10.1186/s42834-021-00080-0.

## Introduction

Water quality is important to safeguard water security and to sustain livelihoods of all living organisms on earth. It is determined by several water parameters, for examples pH, oxidation-reduction potential (ORP) and dissolved oxygen (DO) concentration, which are essential in water quality surveillances and water treatment processes [1, 2]. The measurement of these parameters is driven by electron density in water. For examples, pH and ORP measurements are correlated due to electron exchange generating a potential difference across an electrochemical cell based on the hydrogen ion concentration (pH) and potential value (*E*) [3]. DO concentration is the measurement of molecular oxygen dissolved in water. Likewise, DO measuring technique using polarographic membrane DO sensor involves redox reaction and potential voltage in order to provide an accurate DO value [4, 5]. Such sensor comprises two electrodes immersed in an electrolyte into which the O_2_ in water sample diffuses through the membrane. Electrons flow in the electrode then reduces the O_2_ into hydroxide ions. This will induce a potential which is proportional to the concentration of OH^−^ ions. As a result, DO concentration can be measured and displayed by the meter. Therefore, these measurement principles evidence that electron density is important in determining the pH, ORP and DO concentration.

Conventional approaches using physical and chemical methods are employed to improve pH, ORP and DO concentration. These methods include advanced oxidation process, catalysis, electrolysis, distillation and reverse osmosis [6]. For example, electrolysis works by introducing redox reaction through electron exchange mechanisms across two electrodes. In this process, pH, ORP and oxygen concentration are changed based on the reduction of O_2_ and oxidation of H_2_ as shown in Eqs. (1) and (2), respectively [7].

1 $$ {\mathrm{O}}_2+4{\mathrm{H}}^{+}+4{\mathrm{e}}^{\hbox{-}}\to 2{\mathrm{H}}_2\mathrm{O} $$

2 $$ {\mathrm{H}}_2\to 2{\mathrm{H}}^{+}+2{\mathrm{e}}^{-} $$

On the other hand, several studies [8–11] also reported that magnetic effect can change the pH, ORP and DO concentration of water. This magnetic effect can be originated from electromagnets or permanent magnets. For instance, magnetic effect with average intensity of 1000–2000 G could increase pH and DO concentration, while also decrease the ORP. Recently, Lee et al. [8] reported that magnetic effect can increase the electron density and DO concentration based on reversed electric motor principle. The finding showed that pH, ORP, DO concentrations in distilled water were changed from 5.14 to 5.54 (+ 7.8%), 461 to 367 mV (− 20.4%), and 6.68 to 6.90 mg L^− 1^ (+ 3.3%), respectively when electron is generated in water due to mechanical motion of water intersecting with the magnetic effect at a perpendicular angle.

There have been several works that report the effects of electron generation on water properties. Chen et al. [12] investigated the properties of plasmonic activated water that were attracted to hot electron. Such ‘electron-doped’ water possessed several unique properties such as reduced hydrogen bonds, lower heat capacity, shorter freezing time, and stronger reductive activity. Moreover, the increase of electron density in water that promotes redox mechanism and electron transfer is promising as this contributes many applications in different fields, for examples deoxyribonucleic acid damage, disease treatment and wastewater contaminant remediation [13–18].

While the magnetic effects on pH, ORP, and DO concentration of water have been previously reported, they were usually inferred based on Lorentz Force theory as well as water cluster and hydrogen bonding characteristics [19–21]. One scope that has not been thoroughly investigated is the correlation of the magnetic effect with electron generation in water which can lead to the improvement of DO concentration. Therefore, this study employs a natural and facile method using permanent magnets to co-generate electron and oxygen in water based on reversed electric motor principle. In order to corroborate the correlation between electron generation and DO concentration, a systematic investigation on water magnetization process through the analyses of water parameters such as pH, ORP and DO concentration was performed under different magnets arrangements and water flow rates. The number of electron generation was calculated through Nernst equation. In addition, the magnetized water was characterized using Raman spectroscopy to indicate the magnetization effect on water properties, for examples, hydrogen bond and water cluster characteristics, which can provide new insights into prospective water science and molecular investigations. Such correlation of electron generation and DO concentration improvement under the influence of magnetic field is essential as it reveals the potential of naturally generating electron and oxygen concurrently from water source without using electricity, unlike electrolysis. With the coexistence of electron and oxygen in water, this green technology can be used in environmental applications such as water treatment and wastewater contaminant remediation, as well as in medical applications including free radical induced-diseases treatment.

## Materials and methods

Deionized (DI) water was used as the water source for the magnetization process in this study. DI water was produced from Milli-Q Academic Ultrapure (Type 1) Water Purification System (Merck Millipore, USA). The pH, ORP and DO concentration of water were measured using STPURE pH electrode (Ohaus), ORP electrode (Hanna Instruments) and optical rugged DO probe (Eutech, Thermo Scientific), respectively. Magnetic effect between 1000 and 1500 G was generated from permanent magnets.

### Design of water magnetization system

**Fig. 1:**
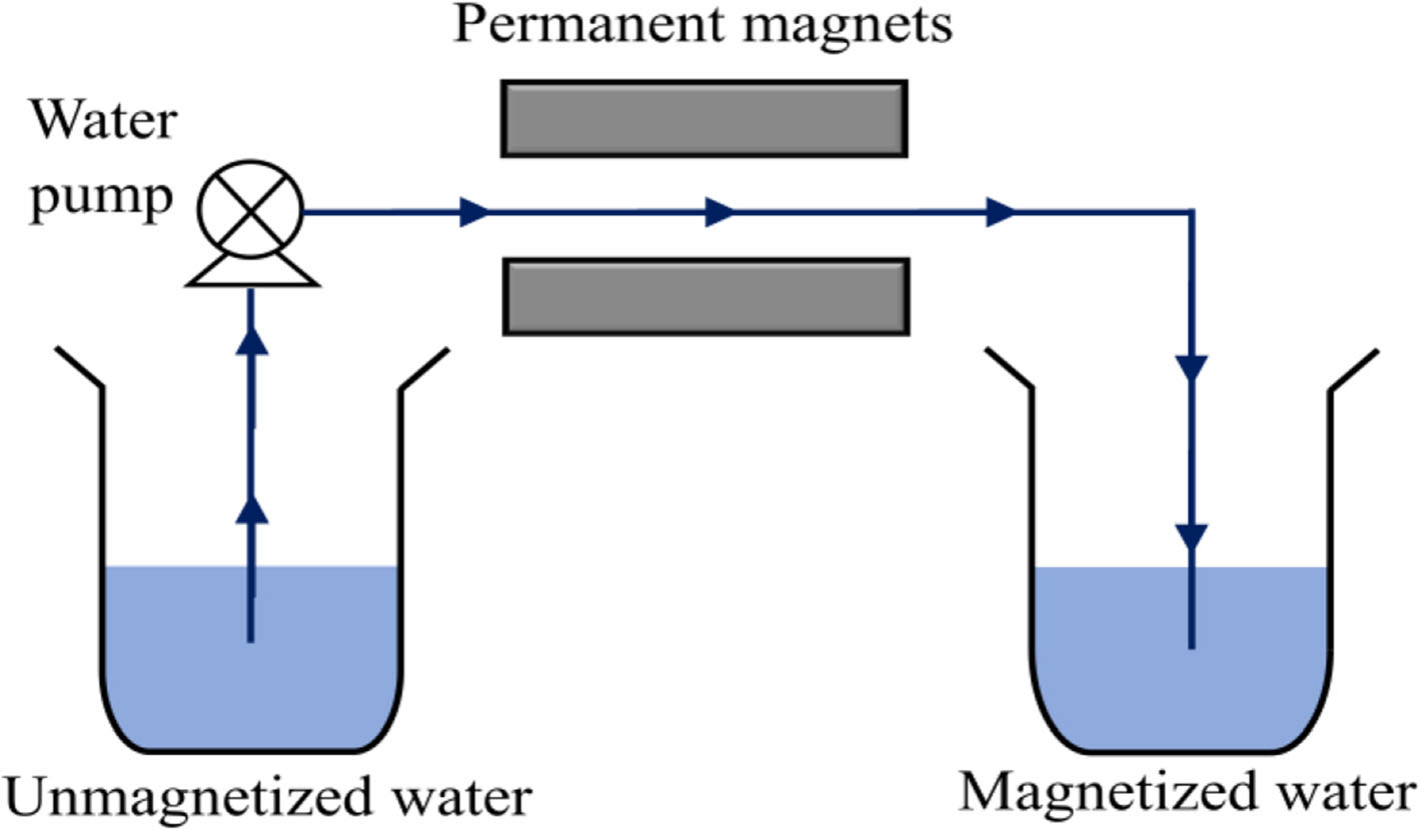
The flow of water through magnetic effect

**Fig. 2:**
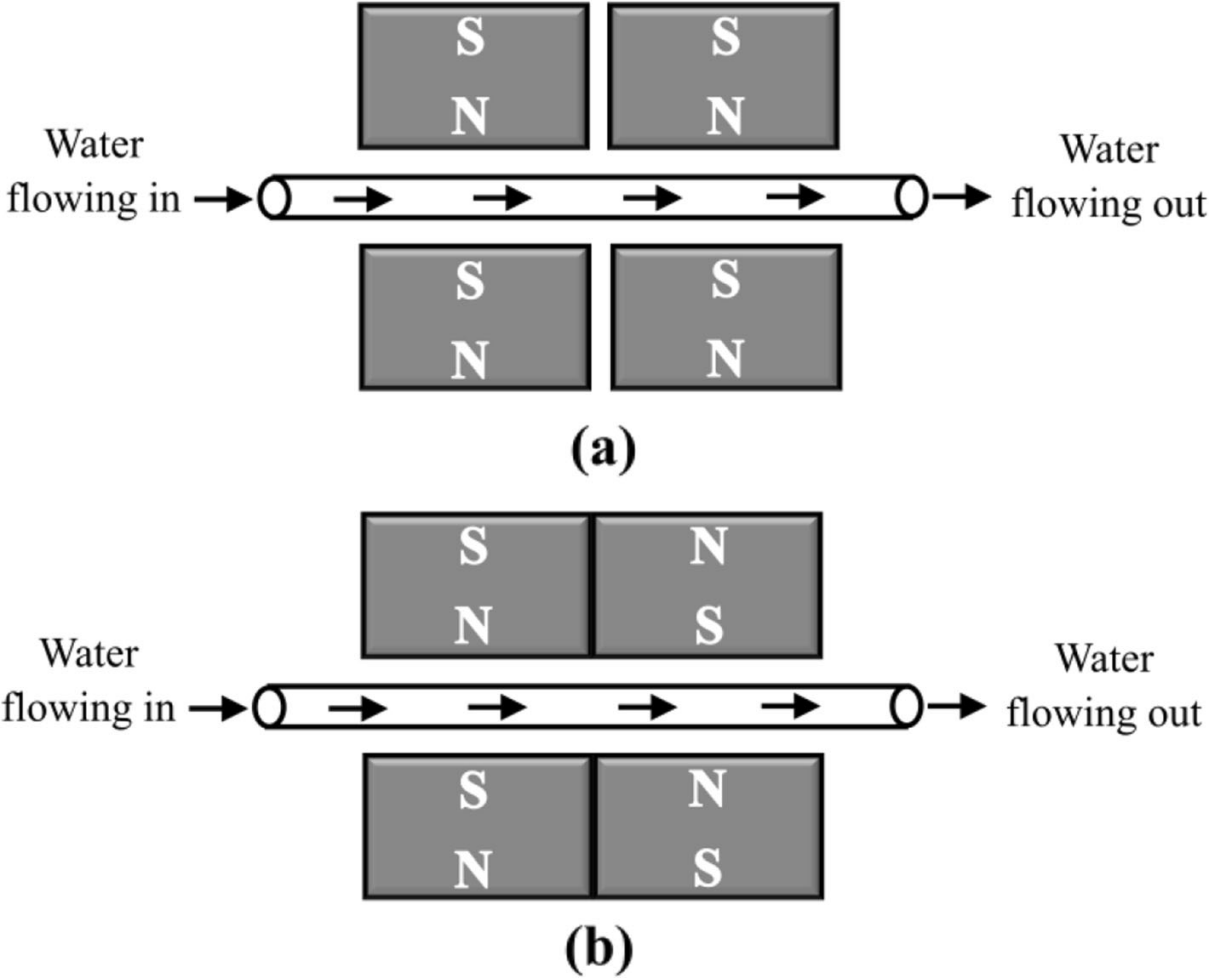
The magnets arrangements used in the study: **a** non-reversed polarity; and **b** reversed polarity

A water magnetization system was designed as shown in the schematic diagram (Fig. 1). The pH, ORP and DO concentration of 100 mL water were measured before and after magnetization. The magnetic effects on water parameters were investigated for variables such as arrangements of permanent magnets and water flow rates. Figure 2 shows the magnets arrangements while Table 1 summarizes the variables and their ranges. Control experiment was performed by flowing the water in the absence of magnetic effect in order to validate the influence of magnetic effects on the water parameters. The changes of water parameters were calculated and reported as percentage change based on Eq. (3).

3 $$ \mathrm{Percentage}\ \mathrm{change}\ \left(\%\right)=\frac{V_a-{V}_b}{V_b}\times 100\% $$

where *V*_a_ and *V*_b_ represent the measured values of water parameters after flowing and before flowing, respectively. A positive percentage change signifies increment of value whereas a negative percentage change signifies decrement of value.

**Table 1:** Experimental variables for the influence of magnetic effect on water parameters

Variables	Range
Water flow rate	Low: 0.1–0.5 mL s^− 1^;
	Medium: 1.0–1.5 mL s^− 1^;
	High: 2.0–2.5 mL s^− 1^
Arrangement of permanent magnets	Non-reversed polarity;
	Reversed polarity

### Characterization of magnetized water using Raman spectroscopy

Both unmagnetized and magnetized water were analyzed by using confocal Raman spectrometer (Renishaw inVia™) integrated with a confocal Raman microscope. The light source used was green laser with excitation wavelength of 532 nm. Laser power was fixed at 5%. Approximately 0.5–1.0 mL of water droplet was placed on top of a silicon disk substrate on the microscope stage. The light source was focused onto the water droplet through a 50*×* long distance lens.

## Results and discussion

### Effect of magnets arrangements on water parameters

**Fig. 3:**
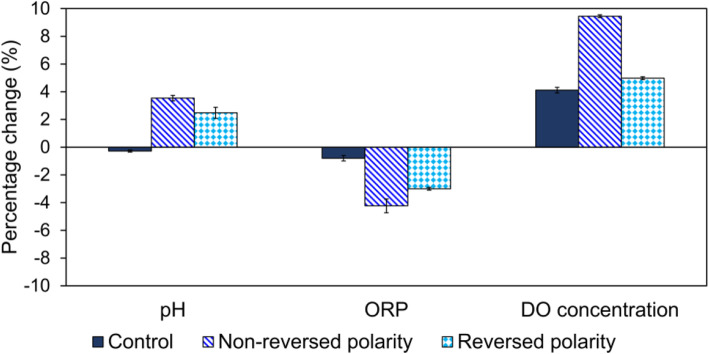
Results of pH, ORP and DO concentration of water after magnetization by using different magnets arrangements

The water flowed under non-reversed polarity magnets arrangement at flow rate of 0.1–0.5 mL s^− 1^ showed an overall better improvement of pH, ORP and DO concentration in comparison to the flow under reversed polarity magnets arrangement. The percentage changes of pH, ORP and DO concentration for this arrangement were + 3.5, − 4.2 and + 9.5%, respectively (Fig. 3; Table S1). On the contrary, the reversed polarity magnets arrangement yielded smaller percentage changes, which were + 2.5, − 3.0 and + 5.0%, respectively (Fig. 3; Table S1). The magnetic effect generated from non-reversed magnetic poles was almost uniform, as compared to its reversed counterpart which generated a highly discontinuous field [22, 23]. Furthermore, magnets with reversed and non-reversed configurations also corresponded to different magnetic field waveforms, with the latter having more homogenous waveform [24]. Nonetheless, Alabi et al. [25] claimed that the purpose of using any magnetic configuration was to generate a uniform magnetic field within the passage of water flow. Hence, most essentially, the orientation of magnets should be in such a way that the water flow is always perpendicular (90°) to the magnetic field lines [26].

### Effect of flow rate on water parameters

**Fig. 4:**
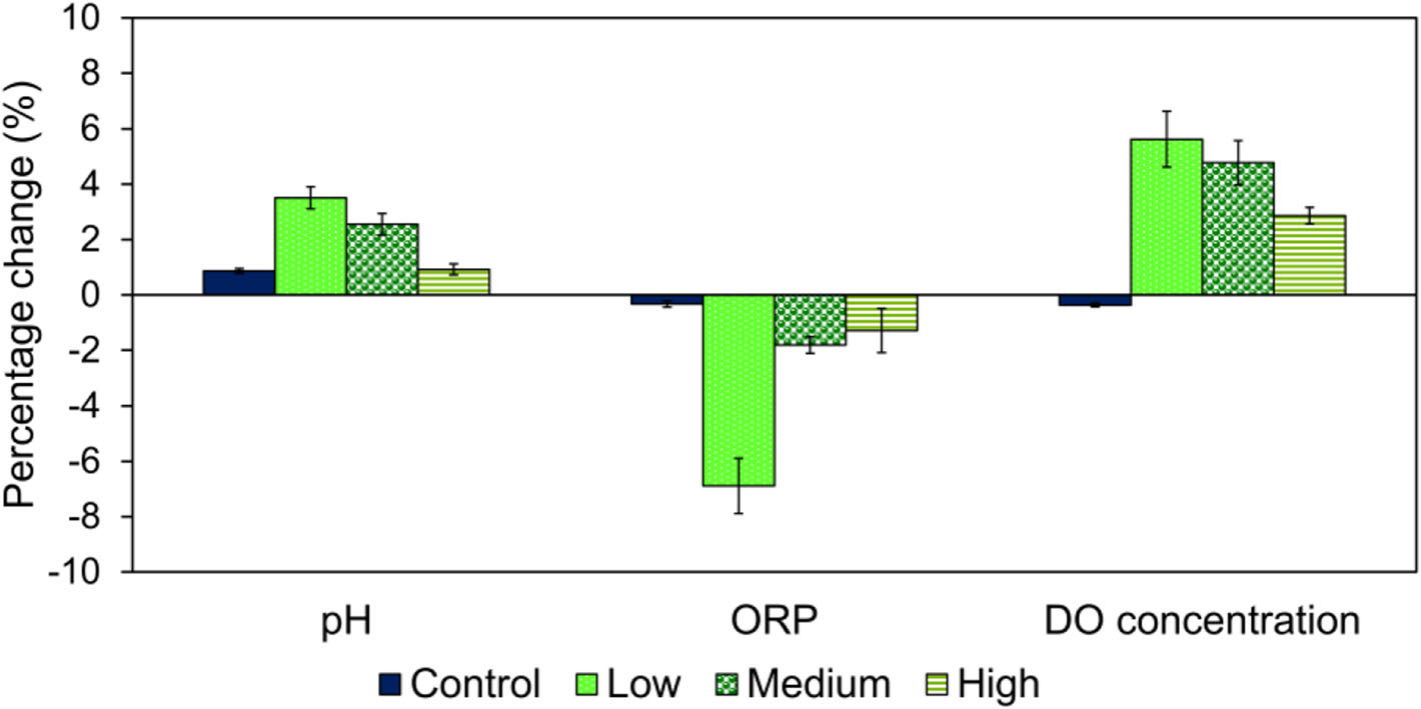
Results of pH, ORP and DO concentration of water after magnetization at low (0.1–0.5 mL s^− 1^), medium (1.0–1.5 mL s^− 1^) and high (2.0–2.5 mL s^− 1^) flow rate, respectively

The results in Fig. 4 and Table S2 revealed that when water flowed through magnetic effect at the lowest flow rate, which was 0.1–0.5 mL s^− 1^, a better improvement of pH, ORP and DO concentration was achieved. In this case, the percentage changes of pH, ORP, and DO concentration after magnetization amounted to + 3.5, − 6.9 and + 5.6%, respectively. When flow rate increased, the percentage changes of water parameters decreased. As shown in Fig. 4, when flow rate increased further to 1.0–1.5 mL s^− 1^, the percentage changes decreased to + 2.6, − 1.8 and + 4.8%, respectively. Subsequently, when the highest flow rate (2.0–2.5 mL s^− 1^) was reached, the percentage changes recorded the lowest, which amounted to + 0.92, − 1.3 and + 2.9%, respectively. Similar findings were reported in the study conducted by Kadhim and Al-Rufaye [27] where the average change in water parameters such as pH, ORP and electrical conductivity of well water decreased from + 1, + 16 and + 3% to 0, + 1 and + 1%, respectively when flow rate increased from 0.1 to 1 L s^− 1^. Besides, the magnetization effect was reported to increase at low flow rate, indicating a longer exposure time of water to magnetic effect [28]. Therefore, these results can show that lower flow rate increases the exposure time of water to magnetic effect, leading to the overall improvement of water parameters.

### Correlation of pH and ORP calculations

Typically, from the experimental findings, when water flowed through magnetic effect, its pH and DO concentration increased whereas its ORP decreased. This trend could be explained through reversed electric motor principle. Primarily, this principle is referenced from the electric motor theory [29] such that when current passes through a conductor coil in a magnetic field, a torque (mechanical force) will be generated which in turn moves the coil. As a result of current passing through the conductor coil, the electron will flow, hence producing electricity. The reversed electric motor principle is precisely the reversed process of electric motor theory, which can also be known as the electric generator principle [30]. In the context of water magnetization, the principle imparts the following attributes to the parameters as represented in Eqs. (4) and (5), respectively [31].

4 $$ {\mathrm{H}}_2\mathrm{O}\leftrightharpoons {\mathrm{H}}^{+}+{\mathrm{OH}}^{-} $$

5 $$ 4{\mathrm{O}\mathrm{H}}^{-}\leftrightharpoons 2{\mathrm{H}}_2\mathrm{O}+{\mathrm{O}}_2+4{\mathrm{e}}^{-} $$

Water molecules ionize to form H^+^ and OH^−^ ions under equilibrium condition based on Eq. (4).

1. Current is induced when magnetic effect is disrupted by the mechanical motion of water flow, which is flowing in the direction perpendicular to the magnetic field.
2. The induced current will promote flow of electron.
3. The generated electron has the tendency to bind with H^+^ cation from ionized water molecule through metastable bonding.
4. Consequently, pH of water increases and the concentration of OH^−^ anion also increases simultaneously.
5. When there is excess OH^−^ anion due to pH increment, the chemical equilibrium is disturbed. As a result, subsequent oxidation of OH^−^ anion occurs to form O_2_, H_2_O and electron (Eq. (5)) in order to restore chemical equilibrium based on Le Chatelier’s principle [32, 33].

The presence of electron in water was indicated by the ORP measurement. The principle of ORP measurement states that when ORP value of water decreases, it signifies the increase of electron density in water. This study highlights the significance of pH and ORP due to the reversed electric motor effect. A correlation between pH and ORP can be determined by using the Nernst equation [34] as shown in Eq. (6).

6 $$ E=E{}^{\circ}+\frac{2.3 RT}{nF}\log \left[{\mathrm{H}}^{+}\right] $$

where *E*, *E*°, *R*, *T*, *n*, *F* and [H^+^] represent the measured potential (ORP value) in V, the standard potential value in V, gas constant (8.31) in J mol^− 1^ K^− 1^, absolute temperature in K, stoichiometric number of exchanged electrons in the reaction, Faraday’s constant (96490) in C mol^− 1^ and hydrogen ions concentration, respectively. The redox activity, in terms of *E* value can be measured by the ORP sensor using the analogy of electrochemical cell when a potential difference (electron transfer) occurs between the inert or indicating electrode (usually platinum) and the silver/silver chloride (Ag/AgCl) reference electrode. In this study, two possible half reactions of redox reactions can be assumed, which are the reduction of AgCl [35] and oxidation of OH^−^ [36]. They are shown in Eqs. (7) and (8), respectively.

7 $$ 4\mathrm{Ag}\mathrm{Cl}+4{\mathrm{e}}^{-}\leftrightharpoons 4\mathrm{Ag}+4{\mathrm{Cl}}^{-} $$

8 $$ 4{\mathrm{O}\mathrm{H}}^{-}\leftrightharpoons 2{\mathrm{H}}_2\mathrm{O}+{\mathrm{O}}_2+4{\mathrm{e}}^{-} $$

Based on electrochemistry theory and the standard electrode potential values [35, 36], the reduction of AgCl in the ORP sensor represents the cathode with *E*° = 0.222 V, whereas the oxidation of OH^−^ from water represents the anode with *E*° = − 0.401 V. Thus, the overall *E*° of the cell (*E*°_*cell*_) is 0.623 V, which is calculated based on Eq. (9) [37].

9 $$ E{{}^{\circ}}_{cell}=E{{}^{\circ}}_{cathode}-E{{}^{\circ}}_{anode} $$

By using *E*° = 0.623 V, the experimental and theoretical *E* values can be calculated based on the measured pH values of water in this study using Eq. (6). By assumption, for every pH increment by 1.00, *E* value or ORP value would decrease by 0.015 V (Table S3; Fig. S1).

### Electron generation in water due to magnetic effect

From the pH and ORP correlation calculation, it is also possible to estimate the number of electrons generated in water. The estimated number of electrons generated in the control and magnetization experiments based on pH and ORP measurements were calculated as shown in Table S4, and the results summarized in Table 2. The obtained results are based on the data from optimized magnetization variables, which is non-reversed polarity magnets arrangement combined with the lowest flow rate (0.1–0.5 mL s^− 1^).

**Table 2:** Estimated number of electron generated in water with magnetic effect based on pH and ORP

	Initial value	After flowing	Delta (Δ) value
Control experiment			
pH	5.40	5.44	0.04
ORP (mV)	403	401	-2
Estimated number of electron	1.5 × 10^14^	1.7 × 10^14^	2.0 × 10^13^
Magnetization experiment			
pH	5.41	5.60	0.19
ORP (mV)	392	365	−27
Estimated number of electron	1.6 × 10^14^	2.4 × 10^14^	8.0 × 10^13^

Table 2 reveals that once water flowed through magnetic effect, pH and ORP values are changed accordingly. The number of electrons could increase from 1.6 × 10^14^ to 2.4 × 10^14^, as compared to control which was from 1.5 × 10^14^ to 1.7 × 10^14^ when there was no magnetic effect. Meanwhile, these data imply that both pH increment and ORP decrement are associated with the number of electrons generated in water. Subsequently, when water is magnetized, the number of electron generation could amount to 8.0 × 10^13^, as compared to the control which could amount to only 2.0 × 10^13^. Therefore, this evidences that pH increment can lead to remarkable increase in electron density and DO concentration in water due to magnetic effect.

### Raman spectral analysis

**Fig. 5:**
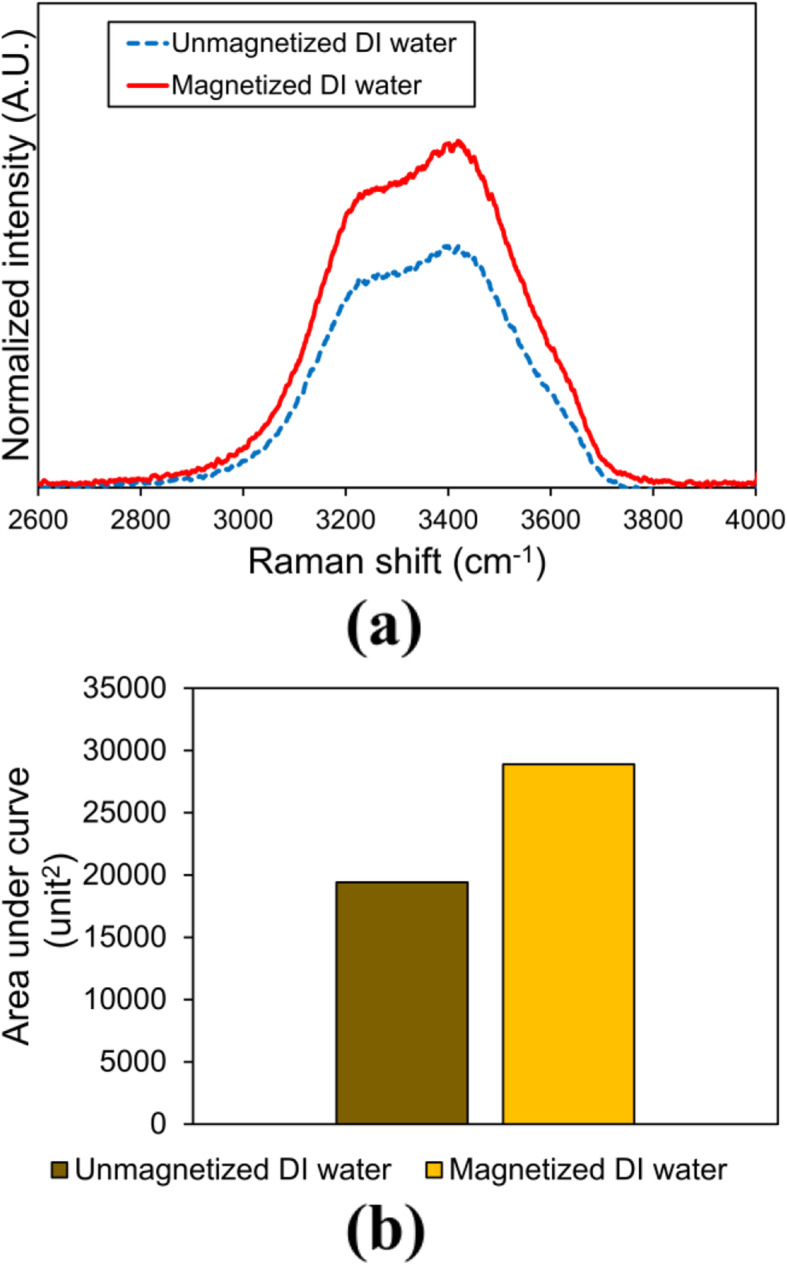
**a** Raman spectra (from 2600 to 4000 cm^− 1^) for unmagnetized DI water, and magnetized DI water produced by water flowing at flow rate of 0.1–0.5 mL s^− 1^; and **b** Computed areas under curve for the unmagnetized DI water and magnetized DI water spectra, respectively

Figure 5a shows the Raman spectra for unmagnetized and magnetized water in the wavenumber of 2600–4000 cm^− 1^. Both the symmetric and asymmetric OH stretching vibrations of water are located between 2900 and 3700 cm^− 1^.

Li et al. [38] stated that the typical Raman spectra of water consists of a peak around 3400 cm^− 1^, an intense shoulder around 3200 cm^− 1^ and a weak shoulder near 3600 cm^− 1^. These typical characteristics are observed in the Raman spectra. As seen in Fig. 5a, magnetized water has an overall greater intensities of the Raman peak and the shoulders than that of unmagnetized water, which aligns with the findings of Pang and Deng [39]. This phenomenon was associated to the alteration in water molecular structures and clusters, as well as the transition of valence, bonded and inner-layer electrons due to magnetic effect [39]. The higher electron density in water generated a higher Raman intensity due to higher electronic transition upon excitation by the electromagnetic irradiation (Raman laser) on water [40].

In addition, a higher Raman OH stretching vibration intensity of water could be associated with stronger intermolecular hydrogen bonds of water [41]. This aligns with the findings of Toledo et al. [19] whereby magnetic effect weakens intra cluster hydrogen bonds of water, which promotes smaller water clusters formation with strong inter cluster hydrogen bonds. Based on the finding of Chen et al. [12], electron played a significant role in the transformation of water clusters and alteration of hydrogen bonds. In particular, water with reduced hydrogen bonds via electron-induced mechanism had a greater peak area of Raman spectra compared to normal DI water. Therefore, the finding in this study, which showed that magnetized water had a greater Raman peak area compared to unmagnetized water (Fig. 5b), aligned with literature findings in terms of generating water with increased electron density, reduced hydrogen bonding and smaller cluster size.

### Implications of study

The electron density and DO concentration increment in water can suggest potential applications in essential water-related industries, particularly in disease control. For examples, most recent literatures have highlighted that free radical damage could have contributed to novel Coronavirus SARS-CoV − 2 infections that cause the severe Coronavirus pandemic alongside other respiratory infection such as the severe acute respiratory syndrome [42]. Besides, cancer can also be attributed to free radical damage induced by reactive oxygen species [43]. These free radical species in body lack electron which makes them highly reactive. As a result, cells are susceptible to oxidative stress. Higher electron density water may be able to counteract the oxidative stress when free radical species scavenge electron from water. In addition, since this study also substantiates DO increment in water due to magnetic effect, it can also be recommended for the treatment of cancers developed from hypoxia (an environment with decreased oxygen level) [44], which is conventionally treated with oxygen nanoshuttles in clinical study [45].

## Conclusions

Magnetic effect increased pH and DO concentration while decreased the ORP of water. The combined variables of non-reversed polarity magnets arrangement and the lowest flow rate (0.1–0.5 mL s^− 1^) led to pH and DO concentration increment by 3.5 and 5.6%, respectively and ORP decrement by 6.9%. These indicate that magnetic effect increases electron density and DO concentration in water. The ORP decrement corresponded to 8.0 × 10^13^ number of electron generation in water. Meanwhile, Raman characterization shows that magnetic effect strengthens intermolecular hydrogen bonding of water molecules and promotes smaller water clusters formation. The electron generation and DO concentration increment in water may be regarded as potential applications in treatment of infectious diseases induced by free radical species.

## Supplementary Information

**Additional file 1.**

## Data Availability

All data generated or analyzed during this study are available from the corresponding author upon request.
