# Supplementary material for: Electron generation in water induced by magnetic effect and its impact on dissolved oxygen concentration
Source: Sustain Environ Res. 2021 Feb 10;31(1):7. doi: 10.1186/s42834-021-00080-0 (PMC7875160; doi:10.1186/s42834-021-00080-0)
Supplement: Supplementary file 1 — Additional file 1. [file 42834_2021_80_MOESM1_ESM.docx]

**Supplementary materials**

**Table S1** Measured values of water parameters before and after magnetization under different magnet arrangements

| Non-reversed polarity magnet arrangements | | | |
| --- | --- | --- | --- |
| Parameters | Before magnetization | After magnetization | Delta value (Δ) |
| pH | 5.65–5.66 | 5.85–5.86 | 0.19–0.21 |
| ORP (mV) | 378 ± 1 | 362 ± 1 | -16 ± 2 |
| DO (mg L^-1^) | 7.30 ± 0.00 | 7.99 ± 0.00 | 0.69 ± 0.00 |
| Reversed polarity magnet arrangements | | | |
| pH | 5.64–5.66 | 5.78–5.80 | 0.12–0.16 |
| ORP (mV) | 366 ± 0.5 | 355 ± 0 | -11 ± 0.5 |
| DO (mg L^-1^) | 7.63 ± 0.005 | 8.01 ± 0.005 | 0.38 ± 0.01 |

**Table S2** Measured values of water parameters before and after magnetization under different flow rates

| 0.1-0.5 mL s^-1^ | | | |
| --- | --- | --- | --- |
| Parameters | Before magnetization | After magnetization | Delta value (Δ) |
| pH | 5.40–5.42 | 5.58–5.62 | 0.17–0.21 |
| ORP (mV) | 392 ± 2 | 365 ± 3 | -27 ± 5 |
| DO (mg L^-1^) | 7.30 ± 0.05 | 7.71 ± 0.02 | 0.41 ± 0.07 |
| 1.0-1.5 mL s^-1^ | | | |
| pH | 5.47–5.49 | 5.61–5.63 | 0.12–0.16 |
| ORP (mV) | 386 ± 0.5 | 379 ± 0.5 | -7 ± 1 |
| DO (mg L^-1^) | 7.32 ± 0.03 | 7.67 ± 0.03 | 0.35 ± 0.06 |
| 2.0-2.5 mL s^-1^ | | | |
| pH | 5.46 | 5.50–5.52 | 0.04–0.06 |
| ORP (mV) | 387 ± 2 | 382 ± 1 | -5 ± 3 |
| DO (mg L^-1^) | 7.35 ± 0.00 | 7.56 ± 0.02 | 0.21 ± 0.02 |

**Table S3** Theoretical *E* values calculated from Nernst equation based on pH 0 to 7 and *E*° = 0.623V

| pH | [H^+^] | log_10_[H^+^] | [OH^-^] | *E*° | Theoretical *E* |
| --- | --- | --- | --- | --- | --- |
| 0.00 | 1.00 | 0 | 1.00 × 10^-14^ | 0.623 | 0.623 |
| 0.50 | 3.16 × 10^-1^ | -0.5 | 3.16 × 10^-14^ | 0.623 | 0.616 |
| 1.00 | 1.00 × 10^-1^ | -1 | 1.00 × 10^-13^ | 0.623 | 0.608 |
| 1.50 | 3.16 × 10^-2^ | -1.5 | 3.16 × 10^-13^ | 0.623 | 0.601 |
| 2.00 | 1.00 × 10^-2^ | -2 | 1.00 × 10^-12^ | 0.623 | 0.593 |
| 2.50 | 3.16 × 10^-3^ | -2.5 | 3.16 × 10^-12^ | 0.623 | 0.586 |
| 3.00 | 1.00 × 10^-3^ | -3 | 1.00 × 10^-11^ | 0.623 | 0.579 |
| 3.50 | 3.16 × 10^-4^ | -3.5 | 3.16 × 10^-11^ | 0.623 | 0.571 |
| 4.00 | 1.00 × 10^-4^ | -4 | 1.00 × 10^-10^ | 0.623 | 0.564 |
| 4.50 | 3.16 × 10^-5^ | -4.5 | 3.16 × 10^-10^ | 0.623 | 0.557 |
| 5.00 | 1.00 × 10^-5^ | -5 | 1.00 × 10^-9^ | 0.623 | 0.549 |
| 5.50 | 3.16 × 10^-6^ | -5.5 | 3.16 × 10^-9^ | 0.623 | 0.542 |
| 6.00 | 1.00 × 10^-6^ | -6 | 1.00 × 10^-8^ | 0.623 | 0.534 |
| 6.50 | 3.16 × 10^-7^ | -6.5 | 3.16 × 10^-8^ | 0.623 | 0.527 |
| 7.00 | 1.00 × 10^-7^ | -7 | 1.00 × 10^-7^ | 0.623 | 0.520 |

**Table S4** Sample calculation of electron generated in water with magnetic effect based on pH and ORP

| Magnetization experiment (Non-reversed polarity and 0.1-0.5 mL s^-1^ flow rate) | | | | | | |
| --- | --- | --- | --- | --- | --- | --- |
|  | pH | pOH ^a^ | [OH^-^] (M) ^b^ | Number of mol of OH^- c^ | Number of moles of electron ^d^ | Number of electron ^e^ |
| Initial | 5.41 | 8.59 | 2.57 × 10^-9^ | 2.57 × 10^-10^ | 2.57 × 10^-10^ | 1.55 × 10^14^ |
| After flowing | 5.60 | 8.40 | 3.98 × 10^-9^ | 3.98 × 10^-10^ | 3.98 × 10^-10^ | 2.40 × 10^14^ |
| Control experiment | | | | | | |
| Initial | 5.40 | 8.60 | 2.51 × 10^-9^ | 2.51 × 10^-10^ | 2.51 × 10^-10^ | 1.51 × 10^14^ |
| After flowing | 5.44 | 8.56 | 2.75 × 10^-9^ | 2.75 × 10^-10^ | 2.75 × 10^-10^ | 1.66 × 10^14^ |

^a^ pOH = 14 – pH

^b^ [OH^-^] = Concentration of OH^-^ = 10 ^-pOH^

^c^ $Number of moles of \mathrm{OH}^{-}\left( in 100 mL water \right)=\left[ \mathrm{OH}^{-} \right]\frac{\mathrm{mol}}{1 L}\times\frac{1 L}{1000 mL}\times100 mL$

^d^ Number of moles of electron = Number of moles of OH^-^ (since mole ratio of OH^-^ to electron = 1:1 according to reaction in Eq. (S1))

${4OH}^{-}\leftrightharpoons{2H}_{2}O+O_{2}+{4e}^{-}$ (S1)

^e^ Number of electrons = Number of moles of electron × *N_A_* where *N_A_* represents Avogadro’s number (6.02 × 10^23^)

**
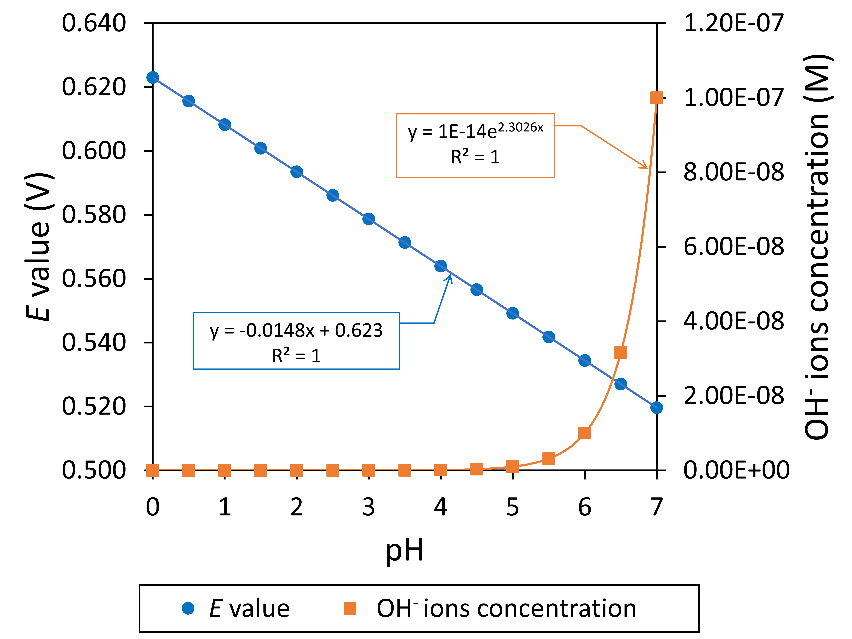
**

**Fig. S1** Relationship between pH and *E* value (ORP) based on theoretical Nernst calculation. The (**•**) plot corresponds to the correlation between *E* value and pH value while (◾) plot corresponds to the correlation between OH^-^ ions concentration and pH value. The equation and *R*^2^ value for both plots are shown.
